# Electroretinography, Ocular Ultrasonography, and Phacoemulsification of Bilateral Cataracts in Two Juvenile Loggerhead Sea Turtles (*Caretta caretta*) of the Mediterranean Region

**DOI:** 10.3390/vetsci10070474

**Published:** 2023-07-20

**Authors:** Andrea Affuso, Barbara Lamagna, Dario Costanza, Dario Basso, Marzia Scarfò, Cristina Di Palma, Claudia Amalfitano, Leonardo Meomartino, Fulvio Maffucci, Sandra Hochscheid, Maria Vanore

**Affiliations:** 1 Marine Turtle Research Group, Department of Marine Animal Conservation and Public Engagement, Stazione Zoologica Anton Dohrn, Via Nuova Macello 16, 80055 Portici, Italy; fulvio.maffucci@szn.it (F.M.); sandra.hochscheid@szn.it (S.H.); 2 Department of Veterinary Medicine and Animal Production, University of Naples “Federico II”, 80137 Napoli, Italy; blamagna@unina.it (B.L.); cristina.dipalma@unina.it (C.D.P.); 3 Interdepartmental Center of Veterinary Radiology, University of Naples “Federico II”, 80137 Naples, Italy; dario.costanza@unina.it (D.C.); meomarti@unina.it (L.M.); 4 Veterinary Clinic Lucrino, 80078 Pozzuoli, Italy; lucrinovet@libero.it; 5 Plaisant S.r.l., Via di Castel Romano 100, 00128 Roma, Italy; marziascarf@yahoo.com; 6 Veterinary Clinic Salus Vet, 80129 Napoli, Italy; claudia.amalfitano@gmail.com; 7 Department of Clinical Sciences, Faculty of Veterinary Medicine, University of Montreal, Saint-Hyacinthe, QC H3C 3J7, Canada; maria.vanore@umontreal.ca

**Keywords:** sea turtle, cataract, phacoemulsification, ocular ultrasonography, electroretinography, reptiles’ eyes

## Abstract

**Simple Summary:**

The present study describes the medical approach to a severe ocular disease impairing the vision of two young sea turtles rescued in the Tyrrhenian Sea. Proper diagnosis and treatment of ocular diseases in these animals is necessary because their sight is extremely important for their survival. Clinical examination of the eyes showed a bilateral lens opacity (cataract). The two turtles underwent an instrumental examination for accurate diagnosis. Ultrasonography was performed to study the anatomical features. Electroretinography was necessary to obtain functional data on the eyes’ ability to translate the light signal perceived by the retina into a nerve signal to be transmitted to the brain. Only after verifying that there were no other eye impairments besides the cataracts, we decided to proceed to remove them surgically. The surgery was performed by phacoemulsification under a microscope. After surgery, both turtles were treated with antibiotics and anti-inflammatory drugs, both parenterally and topically. After 10 months of follow up, the two turtles are able to feed normally and to interact with obstacles. Clinically, all four eyes appear to be healthy.

**Abstract:**

Bilateral cataracts were diagnosed in two rescued juvenile, immature loggerhead sea turtles (*Caretta caretta*), weighing 1.65 and 1.7 kg. Both animals showed vision impairment and difficulty in feeding without assistance. In fact, they did not notice the presence of the food in the tank unless it was brought close to touching the mouth. Ocular ultrasonography and electroretinography showed no lesions of the vitreal body and retinal layer, therefore, both animals were candidates for bilateral cataract surgery. Topical administration of tropicamide + phenylephrine alternating with rocuronium resulted in only minimal mydriasis. Administration of intracameral rocuronium did not improve mydriasis. Phacoemulsification using a one-handed technique was performed bilaterally with a phacoemulsification device (Sovereign, AMO (Abbott Medical Optics^®^). After surgery, the systemic anti-inflammatory drug (dexamethasone 0.2 mg/kg, IM daily for one week) and antibiotics (enrofloxacin 10 mg/kg IM q 72 h, for 4 weeks; ceftazidime 20 mg/kg IM q 72 h for 3 weeks) were administered. Topical ofloxacin, flurbiprofen and tobramycin/dexamethasone were instilled TID for 4 weeks. Both turtles regained vision in both eyes. Results at a 10-month follow-up were satisfactory. This is the first report of cataracts in turtles rescued in the Mediterranean Sea and the first description of surgical treatment of cataracts in loggerhead turtles so young.

## 1. Introduction

Sea turtles have been employed as a flagship species for conservation due to several considerations. Historically, they played important roles for humans, especially because they served as symbols, representing icons in different societies and cultures worldwide [1]. They have survived on Earth for millions of years, adapting to significant climatic and environmental changes, thanks to their ability to evolve in shape [2] and in anatomical and physiological characteristics, which allow them to interact optimally with the marine environment [3]. Their extraordinary ability to survive geological environmental changes is also related to their ability to follow different evolutionary strategies [4], and finally to their remarkable resistance to diseases.

Loggerhead sea turtles (*Caretta caretta*), which live in the Mediterranean Sea, are extremely vulnerable, due to numerous threats such as human activities, climate change, and invasion of alien species, in combination with overfishing, pollution, and eutrophication [5,6]. It is described that coastal pollution and, in general, degraded water quality, have a relevant impact as a likely factor in declining turtle health [7], by affecting several biological functions, including vision.

Vision is recognized as the primary sense to obtain food in loggerhead turtles. The Foraging is essential for the animal’s survival, growth and reproductive success [8]. Therefore, diagnosis and treatment of ocular disease in rescued animals becomes an absolute priority for the conservation of this species.

In the 2014, a group of Turkish scientists detected ocular dysfunctions in 37.5% of sea turtles, with cranium injuries, that underwent a systematic ophthalmological examination. They assessed that, due to deep orbits and tight attachment between the palpebral conjunctiva and sclera, loggerhead eyes present difficulties for a complete ocular examination. The difficulty can be increased by Blepharospasm and fibrous ocular discharge, which are often seen in rescued specimens. Therefore, many signs of ocular dysfunction may escape superficial observation [9]. This condition could also partially explain the low number of reported cases of cataracts in loggerheads.

Cataracts are a loss of transparency of the lens cortex, nucleus, or capsule and can vary in size and location within the lens [10].

Although the exact etiologies of the different types of cataracts are not entirely clear, numerous possible causes have been described, including hereditary, inflammatory, degenerative, metabolic, and traumatic factors [11,12].

The only effective treatment for cataracts is surgery [13] and phacoemulsification is the accepted gold surgical standard in veterinary ophthalmology [14,15,16,17].

The phacoemulsification, offers the advantage of a rapid lens removal through a small incision in the limbus or cornea. The lens material is fragmented and suctioned out of the eye while the globe is continuously inflated [13].

Cataract surgery in veterinary patients has had tremendous progress in recent years in terms of surgical technique, ocular pharmacology, and the availability of anti-inflammatory agents, viscoelastic agents, and phacoemulsification. Despite these dramatic changes, adequate preparation of the eye and patient for surgery is essential to avoid complications. Preoperative use of anti-inflammatory agents, preoperative and intraoperative mydriasis, proper positioning of the patient, use of magnification, and use of ophthalmic viscosurgical devices (viscoelastics) are essential factors. Postoperative management is also very important [13].

In dogs, postoperative uveitis is expected following phacoemulsification and for this reason the use of topical anti-inflammatory medications is strongly recommended [13].

In a retrospective study, an incidence of 16.2% of uveitis following phacoemulsification was described in dogs [13,18]. Data on postoperative complications after phacoemulsification in reptiles are still insufficient.

Until a few years ago, the development of cataracts in sea turtles was considered a rare event, and there was only one description of cataract surgery in a loggerhead sea turtle recovered in Virginia [19]. However, in the last decade, a remarkable increase in diagnosed cases of cataracts in loggerhead sea turtles has been reported in animals recovered in North and South Carolina. In addition, a series of ten cases have been described in which treatment with phacoemulsification was performed [20].

In previous reports, authors described clinical cases of sea turtles with body weights ranging from 37 to 80.5 kg. All turtles appeared to have improved vision after the surgery, and all were released; maximum follow-up was two years after surgery [19,20].

The present study describes the diagnostic features and successful surgical treatment of bilateral cataract in two juvenile loggerhead sea turtles.

To our knowledge, this is the first report of cataracts in turtles rescued in the Mediterranean Sea and the first description worldwide of surgical treatment of cataracts in loggerhead turtles performed in such young patients.

## 2. Materials and Methods

### 2.1. Case Presentation

Two juvenile loggerhead sea turtles: Turtle S (TS) and Turtle A (TA), were rescued in the Tyrrhenian Sea.

TS was rescued floating near Cape Miseno, in the north of Naples, in April. As the turtle was equipped with a subcutaneous tag, it was possible to identify and contact the rescue center that had released it, which made it possible to determine its exact date of birth. The TS egg was sampled from the nest in Barcelona on 15 July 2020, and it incubated near the Rescue Centre for Marine Animals (CRAM) in Barcelona. On 5 September 2020, the egg hatched, and TS was then reared in captivity until July 2021, as part of a head start program [21] conducted by CRAM. The CRAM scientists declared that TS did not present cataracts during the year it was at the center, although some of its littermates from the same nest presented cataracts that were self-limiting and absorbed, allowing them to be released. A microchip was implanted subcutaneously, prior to release.

Upon arrival at Turtle Point (TP), the Research Center for Large Marine Vertebrates of the Stazione Zoologica Anton Dohrn, TS was 1.8 kg in body weight (BW) and had a 23 cm Curve Carapace Length standard (CCLst). TS appeared to be thin and had mild sensory depression, but overall was not in dramatically poor general clinical condition. Blood parameters were within the range reported for the species [22].

No relevant alterations were noted on general clinical examination, except for the scute pattern, presenting six lateral scutes on the right instead of five.

Radiographic examination revealed the presence of a subcutaneous chip tag in the area between the neck and left shoulder.

TA was not tagged, thus it was not possible to know the history of the turtle, or to determine the hatching date. However, comparing the body weight and biometric data with those of TS, we can assume that it was born in the summer of 2020, similar to TS. TA was rescued adrift near Salerno in conditions similar to TS and, upon arrival at the TP, measured 1.63 kg BW and 22.5 cm CCLs.

Each turtle was kept in a rehabilitation tank (1500 L) belonging to a recirculating aquatic system consisting of three tanks connected to a dedicated Life Support System (LSS), equipped with a mechanical sand filter, a biological moving bed filter, a foam fractionator with ozone injection, a UV treatment unit and a titanium heater for temperature control (22–24 °C). Temperature, pH, Redox potential and salinity were measured daily using a multiparameter probe (model HQ40D, Hach Lange SRL). Nitrates, nitrites, ammonium and phosphates were measured weekly using a portable spectrophotometer (model DR1900, Hach Lange SRL).

At the time of the first food administration both turtles could not recognize it, and were unable to calculate the correct distance for the bite. They were unable to take food when it was away from the mouth (Video S1 and S2 .mp4).

### 2.2. Ophthalmic Examination

A complete ophthalmologic examination was performed. In both turtles, the pupillary light reflexes were reduced on both sides and the menace response was absent, while dazzle responses were present.

**Figure 1:**
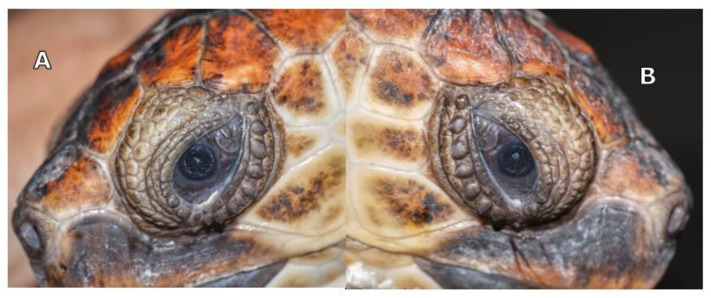
Turtle S: mature cataract of left eye (**A**) and Turtle A: mature cataract of right eye (**B**).

Slit lamp biomicroscopy (Kowa SL-15; Kowa Company Ltd., Tokyo, Japan) showed mature cataracts in both eyes (OU) of the animals. Tonometry using a rebound tonometer (TonoVet^®^, Icare Finland Oy, Vantaa, Finland) revealed IOP values of 9 and 12 mm Hg on the right and left eye, respectively, for TS, and 10 and 12 mm Hg for TA. Indirect ophthalmoscopy was not possible due to the bilateral mature cataracts (Figure 1A,B).

The definitive diagnosis was mature cataracts OU in both animals. Cataract surgery with phacoemulsification was planned. B-scan ocular ultrasonography and electroretinography were planned before phacoemulsification.

### 2.3. Ultrasonography

**Figure 2:**
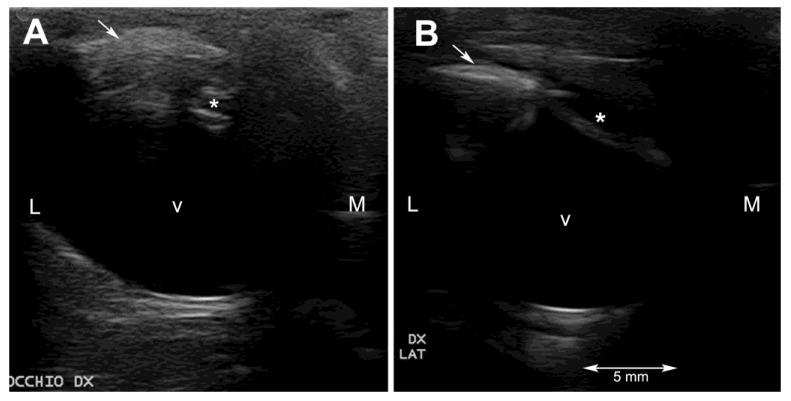
Ultrasonographic horizontal scan of the right eyeball of subject 1 (Alessia) before (**A**) and six months after the surgery (**B**). At the last ultrasonographic exam, the lens appears hypoechoic but thinner than normal. Legend: * = lens; arrow = scleral ossicle; v = vitreal body; L = lateral; M = medial.

**Figure 3:**
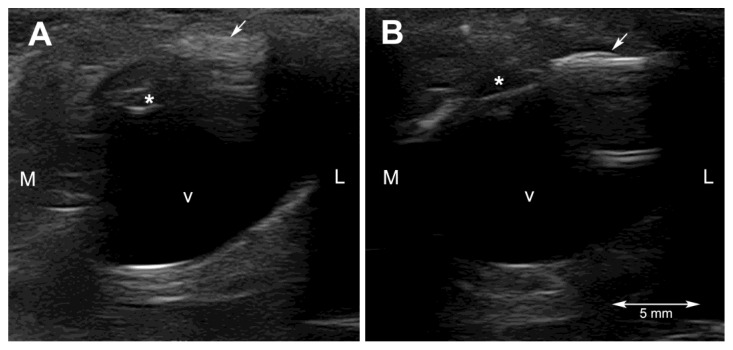
Ultrasonographic horizontal scan of the left eyeball of subject 2 (Scricciolo) before (**A**) and six months after the surgery (**B**). As for subject 1, at the last ultrasonographic exam, the lens appears hypoechoic but thinner than normal. Legend: * = lens; arrow = scleral ossicle; v = vitreal body; L = lateral; M = medial.

Ultrasonography was performed on sedated turtles. The sedation was obtained by administering IM 0.05 mg/kg medetomidine chlorhydrate (Dormilan 1 mg/mL—ATI srl, Italy). Ultrasonography (US) examination was performed using a general purpose US device (MyLab Class C, Esaote, Genova, Italy) equipped with a high frequency linear probe (14 MHz), connected to the cornea through a copious amount of sterile gel (Sterile Aquasonic 100, Parkers Lab., Fairfield, NJ, USA) as previously described [23]. Despite the small size of the eyeballs of these two turtles and the thinner anterior chamber and smaller lens, characteristic of this species, it was possible to visualize all the anatomical structures of the eye. In particular, the lens was echoic instead of the normal anechoic pattern (Figure 2A and Figure 3A).

### 2.4. Electroretinography

Pupillary dilation was attempted by topical application of tropicamide and phenylephrine eye drops and rocuronium bromide as follows: one hour before the procedure, a 20 μL drop of rocuronium bromide 1% and a 20 μL drop of phenylephrine/tropicamide (10% and 0.5%, respectively) were placed in each eye at 2-min intervals. Pupil diameter was measured with a Schirmer tear test strip before administration of the drops (T0), and after 60 min (T60). At T0 pupil diameters were 0.8 mm in both eyes of both turtles. At T60 the pupil diameter increased from baseline in both animals and was 1.5 mm OU.

Electroretinography (RETevet^TM^ LKC TECHNOLOGIES, Gaithersburg, MD, USA) was performed under anesthesia with intramuscular injection of a combination of medetomidine (Dormilan 1 mg/mL—ATI Srl, Erba, Italy), at a dose of 0.05 mg/kg, and ketamine (Lobotor 100 mg/mL–Acme Srl, Corte Tegge, Italy) at a dose of 2.0 mg/kg.

After administration of premedication, each turtle was kept in a dark room for 20 min to acclimate to the darkness. Then, anesthetic eye drops (oxybuprocaine hydrochloride 0.4%; Novesina, Novartis farma spa, Origgio, Italy) were instilled into both eyes and electrodes were placed, under the illumination of a red-light LED flashlight (620 nm).

**Figure 4:**
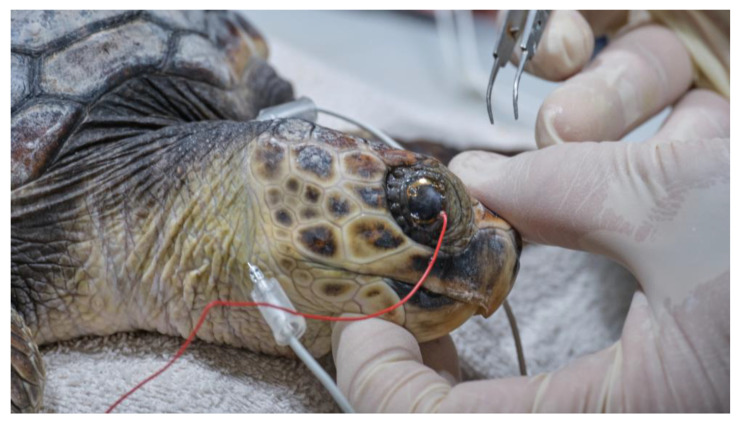
Electrode application for the ERG.

The electrodes were placed using a variation of the method used in dogs. The recording electrode was in a contact lens (ERG-jet, diameter 12 mm) that was placed on the cornea, after covering half of it with gonio gel (idrossipropilmetilcellulose 2%). The reference electrode (needle 12 mm × 29 gauge) was placed subcutaneously, behind the lateral canthus on the neck, due to the difficulty of introducing the needle to the facial skin covered with scales. Similarly, the ground electrode (needle 12 mm × 29 gauge) was placed subcutaneously in the center of the dorsal neck (Figure 4).

The protocol ECVO 5-step single flash, one of the protocols recorded in the RETevet^TM^ device, was used. It is based on the guidelines for testing dogs, derived from the basis established by the European College of Veterinary Ophthalmologists.

*Scotopic ERGs,* for assessing the functioning of the rod pathways in the retina, were obtained by using three single flashes: 0.01 (0.01 cd·s/m^2^ @ 0.2 Hz), 3.0 (3 cd·s/m^2^ @ 1/15 Hz), and 10.0 (10 cd·s/m^2^ @ 0.05 Hz). Next, after 10 min of light adaption, photopic ERGs, for assessing the functioning of the cone pathways, were acquired using two flash protocols: 3.0 (3 cd·s/m^2^ @ 2 Hz; 20 flashes) and 3.0 flicker (3 cd·s/m^2^ @ 28.3 Hz; 141–424 flashes) after 10 min of light adaptation.

**Figure 5:**
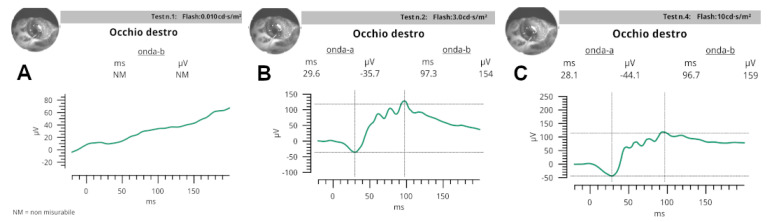
Scotopic ERGs, for assessing the functioning of the rod pathways in the retina, obtained by using three single flashes: 0.01 (0.01 cd·s/m2 @ 0.2 Hz) (**A**), 3.0 (3 cd·s/m2 @ 1/15 Hz) (**B**), and 10.0 (10 cd·s/m2 @ 0.05 Hz) (**C**).

In scotopic ERG (Figure 5) at 0.01 cd·s/m^2^, b-wave amplitude and peak time were NM in ODX and OSN of turtle S; NM in ODX and OSN in turtle A.

At 3.0 cd·s/m^2^: median a-wave amplitude and peak time values were −35.7 (−33.5; −53.6) μV and 31.6 (29.6; 35.4) ms, respectively; median b-wave amplitude and peak time values were 142 (59.1; 154) μV and 97.3 (49.1; 103.4) ms.

At 10.0 cd·s/m^2^: median a-wave amplitude and peak time values were −44.1 μV (22.1; 47.1) and 28.1 (26.5; 31.3) ms, respectively; median b-wave amplitude and peak time values were 194 (159; 222) μV and 102.8 (96.7; 110.9) ms, respectively.

In photopic ERG, at 3.0 cd·s/m^2^ a-wave amplitude and peak time were in turtle S: ODX NM and OSN NM; in turtle A: ODX NM and OSN NM.

At 3.0 flicker median values recorded were 6.5 μV (6;12.4) and 47.2 ms (45.8; 47.3).

### 2.5. Pre-Surgical Preparation and Phacoemulsification Technique

Turtles received topical antibiotics (tobramycin, Tobramycina^®^ and ofloxacin, Exocin^®^) and a topical non-steroidal anti-inflammatory drug (flurbiprofen, Ocufen^®^) three times daily for one week in both eyes as preoperative treatment. A physiologic saline was applied to both eyes three times daily, three minutes prior to topical treatment administration, to remove the dense mucoid ocular discharge naturally produced in this species and to promote tissue penetration of the drugs.

To achieve mydriasis before the anesthesia induction, two drops of rocuronium bromide (10 mg/mL) and 10% phenylephrine combined with tropicamide (VISUMIDRIATIC^®^) were applied topically to both eyes six times at 2-min intervals between each other.

**Figure 6:**
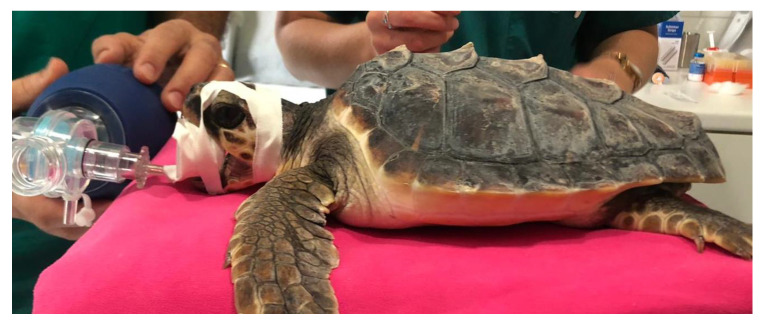
Surgery preparation: The turtle was intubated; the tracheotube was fixed with adhesive plaster strips and protected by tubular plastic mouth opener.

For the anesthesia, the initial sedation was obtained by intramuscular injection of a combination of medetomidine (Dormilan 1 mg/mL—ATI srl, Italy), at the dose of 0.05 mg/kg, and ketamine (Lobotor 100 mg/mL–Acme Srl Italy) at the dose of 2.0 mg/kg. Around 15 min later, induction was obtained through intravenous injection of Propofol (Propsure—Merial Italia SpA, Noventana, Italy) at a dose of 5 mg/kg. After the induction, the turtles were intubated with a PVC disposable ID 3 endotracheal tube (Teleflex Medical S.r.l Via Torino 5 20814 Varedo-MB). The tracheal tube was fixed with strips of adhesive plaster and protected with a mouth opener sheath (Figure 6). After intubation the gas anesthesia was maintained with Isoflurane, in a range from 2.0 to 0.5% (Isoflo Zoetis Italia srl). The heart rate was monitored by a doppler device, and a cloacal probe was used to evaluate the temperature. The turtles required a positive pressure ventilation during the general anesthesia.

During surgery, the turtles were placed on the surgery table in decumbency on the plastron. The neck was rotated to have the eye to be approached facing upwards. The surgeon was positioned laterally to the turtle’s head. The ocular surface was cleaned with 5% dilute povidone iodine and physiologic saline.

In order to obtain a complete cornea exposure, avoiding the globe retraction when the conjunctiva was grasped with forceps, topical anesthesia was attempted with 2% lidocaine. However, the globe retraction persisted even when the turtle was in a deep plane of anesthesia, as reported by Westermeyer. Intracameral atracurium (0.1 mL, 1:10 dilution in a balanced salt solution) was administrated into the anterior chamber before surgery, without any significant effect on mydriasis.

**Figure 7:**
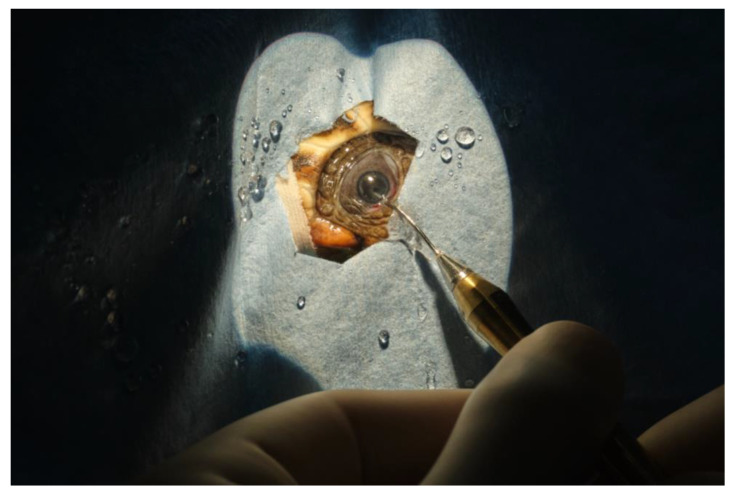
Subcapsular hydro-dissection.

**Figure 8:**
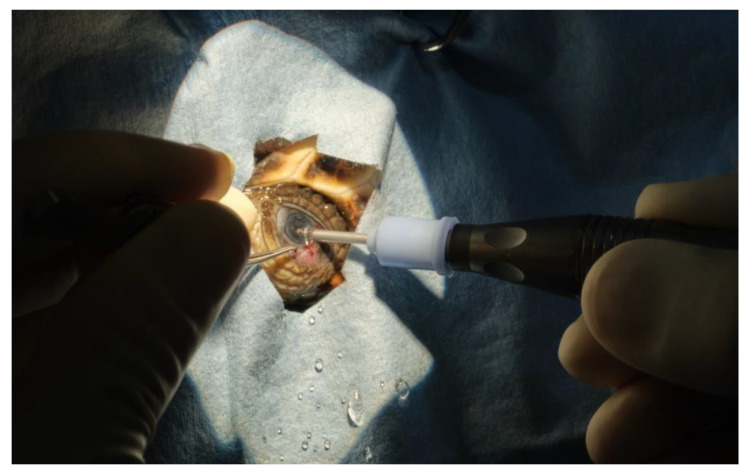
Phacoemulsification phase.

A full thickness stab corneal incision of 2.85 mm was made with a keratotome. The horizontal corneal diameter (limbus to limbus) was 5 mm in length. The corneal incision was performed 2 mm away from the limbus, to avoid the extremely shallow anterior chamber space near the limbus. Following the corneal incision, the anterior chamber was filled with a viscoelastic device (Healon pro10 mg/mL sodium hyaluronate Johnson & Jonson vision^®^). This resulted in sufficient pupillary dilation to achieve an adequate anterior chamber expansion, to visualize the anterior lens capsule and to perform the anterior capsulotomy. A continuous curvilinear capsulorhexis using Utrata forceps was performed, and the anterior capsule was detached and removed. Subcapsular hydro-dissection has been performed using physiologic saline, 1 mL syringe, and a 28 G needle (Figure 7). In case 1 (TS), the cataract of the right eye was removed at first using mostly irrigation and aspiration without success and short pulses of phacoemulsification power were needed to completely remove the cataract. The phacoemulsification system (Sovereign, AMO -Abbott Medical Optics^®^) was equipped with a 19 G straight needle with a 45° tip (Figure 8). In case 2, lens material was removed by phacoemulsification directly. In both cases, automated irrigation and suction were used to remove the remaining cortical material. Due to poor pupillary dilation, this had to be performed without visualization of the instrument tip behind the iris. Between every surgical step of the procedure, a pupillary dilation was obtained by the application of viscoelastic, to observe the integrity of the lens bag and the residual presence of the lens material. The corneal incision was closed by four stitches in a simple interrupted pattern using 8-0 polyglactin 910 resorbable sutures. The ocular surface was routinely cleansed with physiologic saline during the surgical procedure, to keep the cornea hydrated and to allow better visualization of the anterior chamber.

For a postoperative period of five days, the turtles were kept out of the tank enclosure. Then they were reintroduced to the rehabilitation tank.

The postoperative topical treatments repeated the preoperative ones and lasted four weeks. In addition, to define the most suitable parenteral antibiotic therapy, bacterial cultures from oculoconjunctival swabs were performed and led to the isolation of *Vibrio harveyi.* Antibiotic susceptibility testing showed response to amikacine, ceftazidime, ciprofloxacin, gentamicin, doxycycline, enrofloxacin, oxytetracycline, sulfamethoxazole-trimethoprim and flumequine. Therefore, according to the microbiological findings, the turtles were treated with ceftazidime intramuscularly (IM) at a dose of 20 mg/kg every 72 h for three weeks and enrofloxacin 10 mg/kg every 48 h (ENROVET^®^) for four weeks. A systemic anti-inflammatory drug, dexamethasone (Dexadreson^®^) was administered at a dose of 0.2 mg/kg, IM daily for one week, and then the dose was reduced to 0.1 mg/kg daily for a further two weeks.

During the dry period the two sea turtles were not fed. In this period supportive care was administered subcutaneously. It consisted of a mix of fluids composed of 40% Ringer lactate, 40% saline solution and 10% of 5% glucose solution, at a dose of 1% BW daily. When the turtles started feeding again, the glucose was discontinued, and fluid therapy was continued for another two weeks.

## 3. Results

**Figure 9:**
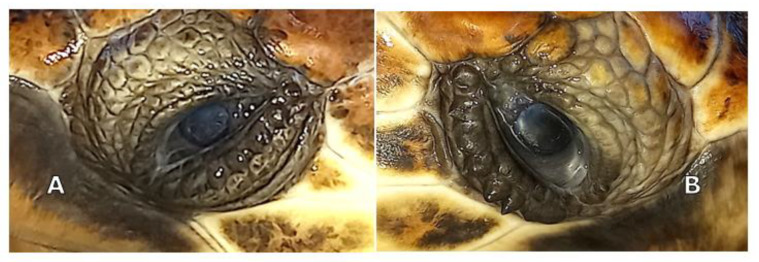
Turtle S: left eye (**A**) and Turtle A: right eye (**B**) three weeks after surgery.

**Figure 10:**
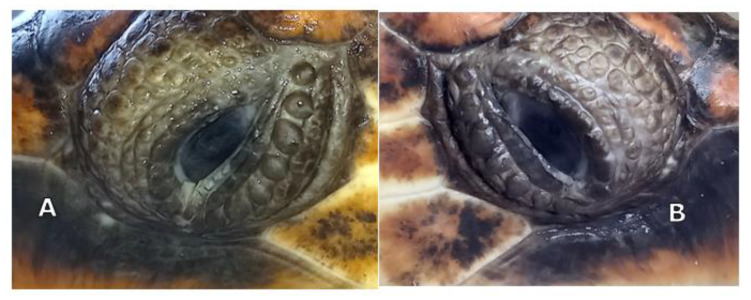
Turtle S: left eye (**A**) and Turtle A: right eye (**B**) ten months after surgery.

The aphakic turtles’ eyes were examined at one week, three weeks, and every month for 10 months after the cataract surgery (Figure 1, Figure 9 and Figure 10). At the first ophthalmic reevaluation, case 1 (TS) presented an IOP of 5 and 7 mm Hg on the right and left eye, respectively, with a positive aqueous flare and ocular discomfort in both eyes. Case 2 (TA) presented an IOP of 7 mm Hg in both eyes, intraocular flare was present without ocular pain in both eyes. In both turtles, mild corneal edema was observed at the incision sites and the stitches were in place.

After 4 weeks, none of the turtles showed signs of intraocular inflammation.

Improvement in vision was examined by observing how the turtles were able to recognize the food scattered in the rehabilitation tank and able to calculate the right distance to bite it. Periodically the ability to interact with obstacles placed into the tank was also tested (Video S3 .mp4).

Ultrasonographically, the turtles were examined one month and 6 months after the surgery. Both animals showed a hypoechoic anterior and posterior chamber without any visible lesions to the eyeballs at the first check, and no changes were seen at the last examination (Figure 2B and Figure 3B).

## 4. Discussion

The peculiar condition of TS, which was held in captivity for the first year after hatching and presented scute pattern abnormalities, induced the TP staff to consider the possibility that the cataract could be related to multi organ developmental anomalies. This condition could be induced by environmental stressors during embryonic development and could be directly or indirectly linked to individual survival, as suggested by some authors [24,25,26]. The hypothesis could be supported by the consideration that in reptiles, abnormal temperatures during gestation or egg incubation are known to influence the occurrence of congenital anomalies [27,28,29].

However, since a second turtle was found that had a similar ocular lesion as TS but no other similarities and no other evidence of potentially congenital abnormalities, the etiology of the disease that was observed should be considered unknown. Several causes are described as being responsible for cataract development, including poor nutrition [30,31], cataractogenic drugs and toxins [32], inflammation due to trauma or infection [11].

In our case, considering the age of the turtles and the initial clinical conditions, poor nutrition or uveitis associated with systemic disease seem to be the most likely, according to the conclusions of Kelly [19].

IOP values, measured by rebound tonometry in these animals affected by bilateral cataract, at the time of diagnosis were not significantly different from values described in healthy animals [33]. Seven days after surgery, IOP values were reduced in both cases, with other associated signs of uveitis. In previous reports IOP was recorded in only one case and measured by applanation tonometry (Tonopen; Mentor Ophthalmics) [34]: the values recorded (14 mm Hg in the right eye and 19 mm Hg in the left eye) were significantly higher, compared with those described in healthy animals using applanation tonometry.

The data reported in this case description provides a useful reference for diagnosing and monitoring the progression of ocular disease in the recovered subjects. A complete ophthalmological examination of Loggerhead eyes is very important and IOP variation is characteristic of various ocular diseases.

Ultrasonography helped to rule out lesions to the vitreal body and to the retinal layer. The ultrasonographic exam allowed visualization of the echoic cataractous lens and its modifications after the surgery. The main difficulties during the ultrasonographic exams were, other than the very small size of the eyeball, reverberation artifacts, resulting from the presence of the scleral ossicles, and the retraction reflex, which is very pronounced in this species.

Electroretinography (ERG) is a noninvasive technique for assessing retinal function in clinical and research ophthalmology. There are several reports of ERG in various wild and exotic animal species. However, few cases have been reported in loggerhead turtles. Only rapid-flicker electroretinography has been used to investigate the photopic (cone-photoreceptor based) visual sensitivities, and only in adults (more than 30 years old) [19]. Furthermore, ERG has been mentioned in adult sea turtles with cataracts but without describing the methods of execution and the values of the measurements obtained [34].

This study represents the first description of an electroretinographic examination in young loggerheads with cataracts. The values obtained may provide a useful reference for clinical evaluations in recovered subjects. The main difficulty was the placement of the electrodes, due to the difficulty in placing the needles on the facial skin covered with scales. Future clinical studies are needed to compare the values obtained with those recorded in healthy subjects. In turtles, retinal atrophy and retinal dystrophies have not been described. It is possible to hypothesize that these pathologies are underdiagnosed, because diagnostic evaluations associated with retinal pathologies are not commonly used in clinical practice. ERG may also be used in toxicological studies as a sensitive method to detect adverse retinal effects [35].

The main problem when performing the surgery was the small size of the eyes and the lenses, and the impossibility of carrying out an adequate mydriasis.

It was reported that all reptile irises are composed of striated muscle exclusively, and neither parasympatholytic nor adrenergic agents would be effective to obtain mydriasis [36]. However, it has been demonstrated that adrenergic agents are required, in conjunction with neuromuscular blockade to achieve mydriasis in turtles [37,38,39,40]. Recently, a combination of topical ophthalmic rocuronium bromide and 10% phenylephrine was effective for mydriasis in juvenile non-sedated loggerhead turtles [41]. In our study we administered 0.5% phenylephrine in combination with 10% tropicamide, this solution is the only ophthalmic formulation available in Italy containing phenylephrine. Our experience demonstrated that this combination, associated with topical and intracameral rocuronium, was ineffective to obtain adequate mydriasis. However, we tested for the first time, topical and intracameral administration of rocuronium bromide in sea turtles under general anesthesia. No local or systemic adverse effects were recorded in treated animals.

Information about the general composition of visual organs and the retinal structures of aquatic organisms in general is incomplete. In the future, additional studies should be performed to evaluate iridial musculature of sea turtles, as well as visual formation stages, retinal structure, and cell type and function, using anatomy, histology, and scanning or transmission electron microscopy.

The improvement in vision and quality of life after the surgery was satisfactory in the cases described here. Postoperative complications included transient uveitis in both subjects during the first month after surgery. In previous phacoemulsification descriptions in sea turtles no postoperative complications were recorded. Clinical studies in a larger number of subjects and with long-term follow-up are needed to clarify the effects of this type of surgical treatment in this species.

## 5. Conclusions

Although wave amplitudes and peak time values could not be recorded in all flash protocols tested, this report demonstrated that ERG is effective in evaluating visual prognosis before cataract surgery in loggerhead sea turtles with cataracts.

The results of this study suggest that phacoemulsification surgery can be successful in juvenile loggerheads and should be considered for vision-compromising cataracts in this species.

We did not consider implanting an artificial intraocular lens (IOL) because it was difficult to obtain a suitable lens in terms of shape and size. In addition, in such young sea turtles it is foreseeable that the eye would increase in size and the lens would likely not fit in the adult and could result in IOL decentration or other complications such as the formation of posterior capsular opacification (PCO) which is quite common in humans and dogs after IOL implantation [42]. As a general consideration, the implantation of artificial prosthetic devices in wild animals is not recommended because it is not possible to continue monitoring the patient after the release back into nature. Our decision is in accordance with what is reported in the literature, given that in previous studies the turtles, following the removal of the lens, were left aphakic [19,20].

Future improvement in our ability to assess the visual ability of aphakic turtles, before reintroduction into the wild, could be achieved by using ERG to quantitatively assess vision.

Given the importance of vision for sea turtle survival in the wild [43], cataracts should be included among sea turtle eye diseases to be carefully investigated. A thorough evaluation of the eye, including an ERG, should be performed, whenever vision impairment is suspected, to improve prognosis and to successfully reintroduce these endangered species to their natural habitat.

## Data Availability

The data presented in this study are available on request from the corresponding author.

## Supplementary Materials

The following supplementary information can be downloaded: https://www.mdpi.com/article/10.3390/vetsci10070474/s1, Video S1 .mp4 Sea turtle swims over food in tank without seeing it; Video S2 .mp4 Turtle tries to grab food but has difficulty calculating distance; Video S3 .mp4 Turtle interacts with obstacle, bites it and avoids it to move on.
